# Clinical Characteristics and Mortality Predictors of Group B Streptococcus Bacteremia in Adults: A Multicenter Retrospective Cohort Study in Japan, 2015–2024

**DOI:** 10.1093/ofid/ofag665

**Published:** 2026-10-09

**Authors:** Kohei Oguni, Hidemasa Akazawa, Shinnosuke Fukushima, Yasuhiro Suzuki, Katsuki Kanai, Yuto Haruki, Kengo Mukuda, Hidemaru Matsumoto, Tatsuya Fujihara, Hideharu Hagiya

**Affiliations:** Department of Infectious Diseases, Okayama University Graduate School of Medicine, Dentistry and Pharmaceutical Sciences, Okayama, Japan; Department of General Medicine, Okayama University Graduate School of Medicine, Dentistry and Pharmaceutical Sciences, Okayama, Japan; Department of Infectious Diseases, Okayama University Graduate School of Medicine, Dentistry and Pharmaceutical Sciences, Okayama, Japan; Department of General Medicine, Okayama University Graduate School of Medicine, Dentistry and Pharmaceutical Sciences, Okayama, Japan; Department of Infectious Diseases, Okayama University Graduate School of Medicine, Dentistry and Pharmaceutical Sciences, Okayama, Japan; Department of Bacteriology, Okayama University, Graduate School of Medicine, Dentistry and Pharmaceutical Sciences, Okayama, Japan; Emergency and Critical Care Center, Kurashiki Central Hospital, Kurashiki, Okayama, Japan; Department of Emergency and Critical Care Medicine, Shimane Prefectural Central Hospital, Izumo, Shimane, Japan; Department of Pharmacy, Tsuyama Chuo Hospital, Tsuyama, Okayama, Japan; Department of Infectious Diseases and General Internal Medicine, Tottori Prefectural Central Hospital, Tottori, Japan; Department of Pharmacy, Japanese Red Cross Society Himeji Hospital, Himeji, Hyogo, Japan; Department of General Internal Medicine, Masuda Red-Cross Hospital, Masuda, Shimane, Japan; Department of General Medicine, Okuizumo Hospital, Okuizumo, Shimane, Japan; Department of Infectious Diseases, Okayama University Graduate School of Medicine, Dentistry and Pharmaceutical Sciences, Okayama, Japan

**Keywords:** bacteremia, group B Streptococcus, invasive group B streptococcal disease, streptococcal toxic shock syndrome, *streptococcus agalactiae*

## Abstract

**Background:**

Invasive group B Streptococcus (GBS) infection in nonpregnant adults is increasing, particularly among older adults and those with underlying comorbidities. However, multicenter data on prognostic factors for adult GBS bacteremia in Japan remain limited.

**Methods:**

We conducted a multicenter retrospective observational cohort study of adults with GBS bacteremia at 7 hospitals in western Japan between 2015 and 2024. Variables analyzed included clinical characteristics, infection sites, microbiological characteristics, and antimicrobial susceptibility profiles. Univariable and multivariable logistic regression analyses were performed to identify factors associated with 30-day mortality.

**Results:**

A total of 442 patients were included. Median age [interquartile range] was 75 [64–84] years, and 83.0% had at least 1 comorbidity. The overall 30-day all-cause mortality for GBS bacteremia was 9.3% (41/442), which rose to 39.3% (11/28) among patients with streptococcal toxic shock syndrome (STSS). In multivariable analysis, STSS was the strongest predictor of death (adjusted odds ratio [aOR], 9.16; 95% confidence interval [CI], 3.28–25.58), followed by immunosuppressive therapy (aOR, 4.62; 95% CI, 1.66–12.86), cirrhosis (aOR, 3.40; 95% CI, 1.23–9.40), respiratory infection (aOR, 3.30; 95% CI, 1.08–10.06), advanced renal dysfunction (aOR, 2.66; 95% CI, 1.09–6.52), and polymicrobial bacteremia (aOR, 2.40; 95% CI, 1.03–5.57), whereas skin and soft tissue infection was associated with lower mortality (aOR, 0.07; 95% CI, 0.01–0.53).

**Conclusions:**

Adult GBS bacteremia carries a high burden of short-term mortality, with a wide variety of clinical presentations, necessitating prompt diagnostic and therapeutic intervention.

## Teaser

In this multicenter Japanese cohort of adults with group B Streptococcus bacteremia, 30-day mortality was 9.3%. Streptococcal toxic shock syndrome was the strongest predictor of death, while immunosuppression, cirrhosis, respiratory infection, advanced renal dysfunction, and polymicrobial bacteremia also worsened outcomes.

*Streptococcus agalactiae* (group B Streptococcus [GBS]) is a commensal bacterium that potentially colonizes the human gastrointestinal and urogenital tracts, and occasionally the upper respiratory tract [1]. Although GBS is a well-recognized cause of neonatal sepsis and meningitis, the incidence of early-onset neonatal disease has substantially declined since the implementation of intrapartum antibiotic prophylaxis [2, 3]. In contrast, the burden of invasive GBS infections appears to be increasing among nonpregnant adults, particularly older adults and those with comorbidities such as diabetes mellitus and obesity [4, 5]. GBS bacteremia is reportedly a major manifestation of invasive GBS disease and is associated with substantial short-term mortality [4].

Common clinical manifestations in adults include bacteremia without focus, skin and soft tissue infections (SSTIs), urinary tract infections (UTIs), and musculoskeletal infections [6]. Recent data from 3 Japanese hospitals have shown that the most common manifestations of invasive GBS disease in older adults are SSTI, bacteremia, and urosepsis, together accounting for more than 3 quarters of all cases [7]. Among musculoskeletal manifestations, GBS has increasingly been recognized as an important cause of septic arthritis [8]. A global meta-analysis estimated an overall case-fatality ratio near 10% for adult invasive GBS disease, with substantial heterogeneity by region [9]. Given its high mortality, identifying prognostic factors is clinically essential to facilitate early risk stratification and optimize the management of high-risk patients. In some cases, invasive GBS infection is complicated by streptococcal toxic shock syndrome (STSS), a rare but life-threatening condition [10]. In a Japanese study, STSS is less commonly caused by GBS than by *Streptococcus pyogenes* (group A Streptococcus), but its mortality rate appears to be high at 30.8% [11]. As invasive GBS disease continues to increase, this severe complication may pose an increasing clinical burden. Moreover, although GBS generally remains susceptible to beta-lactams, increasing resistance to macrolides and clindamycin has become a growing concern, and isolates with reduced penicillin susceptibility have also been reported worldwide [12, 13]. These resistance patterns may influence empirical antibiotic selection, particularly in patients with severe conditions and beta-lactam allergy.

Despite the increasing clinical burden of invasive GBS diseases, data on adult GBS bacteremia in Japan remain limited. Previous Japanese clinical studies of invasive GBS diseases have been conducted at single or a few healthcare centers, which have included relatively limited numbers of patients [5, 7]. Nationwide studies have primarily focused on microbiological and molecular epidemiology, and patient-level predictors of mortality have not been extensively evaluated [14–16]. Therefore, the clinical characteristics and independent predictors of short-term mortality specifically among adults with GBS bacteremia have not been well defined. To address this knowledge gap, we conducted a multicenter retrospective cohort study to delineate the clinical features of adult GBS bacteremia and identify independent risk factors associated with 30-day mortality.

## METHODS

### Study Design and Setting

We conducted a multicenter retrospective observational cohort study of patients with GBS bacteremia at 7 hospitals in Okayama, Hyogo, Shimane, and Tottori prefectures, Japan, between January 2015 and December 2024. Characteristics of the participating hospitals are summarized in Supplementary Table 1. The study protocol was approved by the Ethics Committee of Okayama University Hospital (No. 2511-007) and the institutional review boards of all participating hospitals. Given the retrospective nature of the study, the requirement for informed consent was waived, and an opt-out approach was used.

### Participants

Eligible patients were adults aged ≥18 years with at least one blood culture positive for GBS during the study period. We excluded patients who were not hospitalized, had recurrent GBS bacteremia (only the first episode for each patient during the study period was included), had insufficient data to ascertain clinical outcomes because of discharge or transfer within 2 days of admission, or lacked detailed clinical information.

### Data Collection

Data were retrospectively extracted from the electronic medical records of each participating institution. Baseline characteristics included age, sex, body mass index (BMI), and residence in a long-term care facility. Comorbidities and host factors included diabetes mellitus, a history of solid or hematologic malignancy, receipt of chemotherapy, immunosuppressive therapy, stroke, dementia, heart failure, liver cirrhosis, advanced renal dysfunction, and pregnancy. Immunosuppressive therapy was defined as ongoing treatment with systemic corticosteroids, regardless of dose, or other immunosuppressive agents at the onset of bacteremia. Advanced renal dysfunction was defined as an estimated glomerular filtration rate (eGFR) < 30 mL/min/1.73 m^2^ at the time of bacteremia onset. Data on infection sites, microbiological findings, treatment, and clinical courses were also collected. Microbiological variables included antimicrobial susceptibility profiles and the presence of polymicrobial bacteremia. Susceptibility testing was performed at each participating laboratory using standard methods, although testing panels varied across institutions. The instruments used for antimicrobial susceptibility testing are listed in Supplementary Table 1. Minimum inhibitory concentrations were interpreted according to Clinical and Laboratory Standards Institute (CLSI) M100, 35th edition (2025), criteria to ensure uniform interpretation across the study period [17]. Treatment and clinical course variables included antimicrobial agents administered, source control procedures (eg, percutaneous or surgical drainage), and intensive care unit (ICU) admission.

### Definitions

Infection sites were determined retrospectively at each participating institution based on clinical, microbiological, and radiologic findings documented in the medical records; site classification was not centrally adjudicated. The infection sites were classified as bacteremia without focus, SSTI, UTI, musculoskeletal infection, cardiovascular infection, respiratory infection, abdominal infection, or other sites of infection. Multiple-site infection was defined as involvement of 2 or more of these infection sites. Hospital-acquired infections were defined as those developing 48 hours or more after hospital admission. Polymicrobial bacteremia was defined as isolation of 2 or more distinct pathogens from the same blood culture set, including GBS. STSS was defined according to the Japanese surveillance criteria as shock accompanied by at least 2 of the following: liver failure, renal failure, acute respiratory distress syndrome, disseminated intravascular coagulation, soft tissue inflammation including necrotizing fasciitis, generalized erythematous rash, or central nervous system abnormalities [18].

### Outcomes and Statistical Analysis

The primary outcome was 30-day all-cause mortality from the index blood culture positive for GBS. Categorical variables were summarized as numbers and percentages and compared using Fisher's exact test. Continuous variables were summarized as medians with interquartile ranges (IQRs) and compared using the Mann–Whitney *U* test. Trends across ordered groups were assessed using the Cochran–Armitage trend test. Survival curves were estimated using the Kaplan–Meier method and compared using the log-rank test. Univariable logistic regression analysis was performed to identify factors associated with 30-day mortality. Variables with *P* < .10 in univariable analysis were considered for inclusion in the multivariable model. The final multivariable logistic regression model was constructed based on clinical relevance and the number of events per variable to minimize overfitting. Multicollinearity was assessed using variance inflation factors; no significant collinearity was identified. ICU admission and surgical drainage were not included in the multivariable model because they were considered intermediate or intervention variables rather than baseline predictors. As a sensitivity analysis, the multivariable logistic regression model was repeated after excluding polymicrobial bacteremia from the model to assess the robustness of the associations between the remaining variables and 30-day mortality. Missing data were not imputed. Univariable analyses were performed using available data for each variable; no missing values were present for variables included in the final multivariable model. A 2-sided *P* value < .05 was considered statistically significant. All statistical analyses were performed using R, version 4.5.2. ChatGPT (OpenAI; accessed March 2026) was used to assist with statistical code development and English-language editing.

## RESULTS

### Clinical Characteristics

A total of 547 cases of GBS bacteremia were identified during the 10-year study period. Of these, 105 cases were excluded for the following reasons: age <18 years (n = 70); non-hospitalized status (n = 15); a second episode in the same patient (n = 13); insufficient outcome data due to discharge or transfer within 2 days of admission (n = 6); and missing detailed clinical data (n = 1). A total of 442 cases of GBS bacteremia were included in the final analysis (Figure 1).

**Figure 1.**
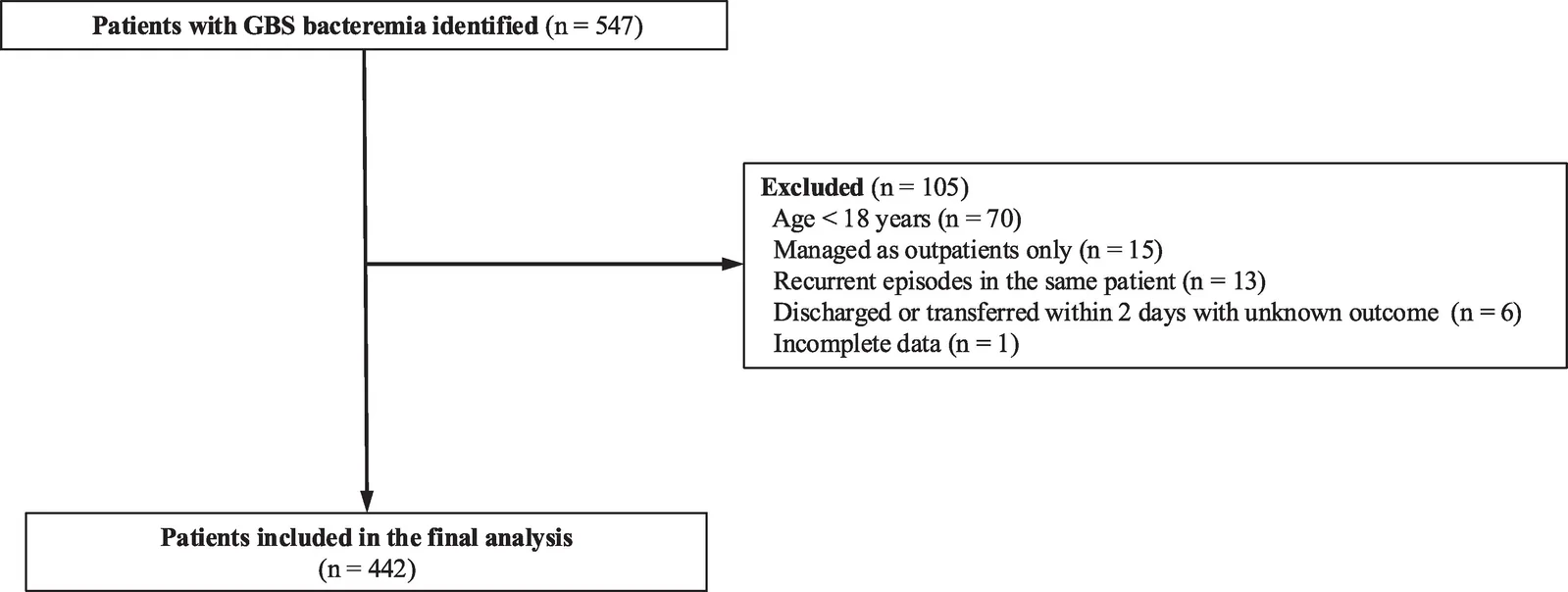
Flow diagram of patient selection. Abbreviation: GBS, group B Streptococcus.

The clinical characteristics of the eligible patients are summarized in Table 1. Overall, the median age was 75 years (IQR, 64–84), and 54% were male. Forty-six patients (10.4%) were residents of long-term care facilities, and 59 (13.3%) were hospital-acquired cases. Underlying medical conditions included diabetes mellitus (38.2%), a history of solid tumors (28.5%), liver cirrhosis (9.7%), advanced renal dysfunction (9.7%), immunosuppressive therapy (7.2%), and obesity (7.2%). Most patients (83.0%) had at least one comorbidity; the median number of comorbidities was 1 (IQR, 1–2), and nearly half had 2 or more comorbidities (47.3%). With respect to the infection foci, bacteremia without focus was most common (120 patients, 27.1%), followed by SSTI in 116 (26.2%), UTI in 96 (21.7%), and musculoskeletal infection in 81 patients (18.3%). The detailed distribution of infection sites is presented in Supplementary Table 2. Multiple-site infections were observed in 42 patients (9.5%). The most common combinations were SSTI and musculoskeletal infection (n = 10), followed by UTI and musculoskeletal infection (n = 7).

**Table 1.** Baseline Characteristics of the Study Cohort According to 30-Day Survival Status

	All Patients (n = 442)	Survivors (n = 401)	Non-survivors (n = 41)	*P* Value
Age (years), median [IQR]	75 [64–84]	74 [64–84]	76 [70–86]	.15
Male sex	240 (54.3%)	218 (54.4%)	22 (53.7%)	1.00
BMI, median [IQR]	22.3 [19.8–25.8]	22.4 [19.8–26.0]	21.7 [19.8–24.2]	.21
Residence in long-term care facility	46 (10.4%)	41 (10.2%)	5 (12.2%)	.60
Hospital-acquired infection	59 (13.3%)	47 (11.7%)	12 (29.3%)	.006^a^
Number of comorbidities				
0	75 (17.0%)	71 (17.7%)	4 (9.8%)	.27
1	158 (35.7%)	149 (37.2%)	9 (22.0%)	.06
2–3	184 (41.6%)	165 (41.1%)	19 (46.3%)	.62
≥ 4	25 (5.7%)	16 (4.0%)	9 (22.0%)	<.001^a^
Comorbidities				
Obesity (BMI ≥30)	30 (7.2%)	29 (7.6%)	1 (2.8%)	.50
Diabetes mellitus	169 (38.2%)	150 (37.4%)	19 (46.3%)	.31
History of malignancy				
Solid tumor	126 (28.5%)	112 (27.9%)	14 (34.1%)	.47
Hematological malignancy	32 (7.2%)	29 (7.2%)	3 (7.3%)	1.00
Chemotherapy	43 (9.7%)	36 (9.0%)	7 (17.1%)	.10
Immunosuppressive therapy	32 (7.2%)	24 (6.0%)	8 (19.5%)	.005^a^
Stroke	56 (12.7%)	51 (12.7%)	5 (12.2%)	1.00
Dementia	51 (11.5%)	46 (11.5%)	5 (12.2%)	.80
Heart failure	78 (17.6%)	66 (16.5%)	12 (29.3%)	.05
Cirrhosis	43 (9.7%)	34 (8.5%)	9 (22.0%)	.01^a^
Advanced renal dysfunction	43 (9.7%)	32 (8.0%)	11 (26.8%)	<.001^a^
Pregnancy	1 (0.2%)	1 (0.2%)	0 (0%)	1.00
Site of infection				
Bacteremia without focus	120 (27.1%)	103 (25.7%)	17 (41.5%)	.04^a^
SSTI	116 (26.2%)	115 (28.7%)	1 (2.4%)	<.001^a^
Cellulitis	95 (21.5%)	94 (23.4%)	1 (2.4%)	<.001^a^
Diabetic foot	8 (1.8%)	8 (2.0%)	0 (0%)	1.00
Necrotizing fasciitis	6 (1.4%)	6 (1.5%)	0 (0%)	1.00
UTI	96 (21.7%)	85 (21.2%)	11 (26.8%)	.43
Pyelonephritis	93 (21.0%)	82 (20.4%)	11 (26.8%)	.32
Musculoskeletal infection	81 (18.3%)	75 (18.7%)	6 (14.6%)	.67
Spondylitis	39 (8.8%)	38 (9.5%)	1 (2.4%)	.16
Septic arthritis	32 (7.2%)	28 (7.0%)	4 (9.8%)	.52
Cardiovascular infection	26 (5.9%)	22 (5.5%)	4 (9.8%)	.29
Infective endocarditis	20 (4.5%)	16 (4.0%)	4 (9.8%)	.10
Respiratory infection	23 (5.2%)	17 (4.2%)	6 (14.6%)	.01^a^
Pneumonia	22 (5.0%)	17 (4.2%)	5 (12.2%)	.04^a^
Pleuritis	1 (0.2%)	0 (0%)	1 (2.4%)	.09
Abdominal infection	12 (2.7%)	10 (2.5%)	2 (4.9%)	.31
SBP	3 (0.7%)	2 (0.5%)	1 (2.4%)	.25
GI perforation	1 (0.2%)	0 (0%)	1 (2.4%)	.09
Others	12 (2.7%)	12 (3.0%)	0 (0%)	.61
Meningitis	5 (1.1%)	5 (1.2%)	0 (0%)	1.00
Endophthalmitis	2 (0.5%)	2 (0.5%)	0 (0%)	1.00
Multiple-site infection	42 (9.5%)	36 (9.0%)	6 (14.6%)	.26
STSS	28 (6.3%)	17 (4.2%)	11 (26.8%)	<.001^a^
Polymicrobial bacteremia	66 (14.9%)	51 (12.7%)	15 (36.6%)	<.001^a^
Percutaneous drainage	32 (7.3%)	29 (7.2%)	3 (7.3%)	1.00
Surgical drainage	58 (13.2%)	57 (14.2%)	1 (2.4%)	.03^a^
ICU admission	98 (22.3%)	77 (19.3%)	21 (51.2%)	<.001^a^

Data are presented as number (%) for categorical variables and median (interquartile range) for continuous variables. *P* values were calculated using the Fisher exact test for categorical variables and the Mann–Whitney *U* test for continuous variables. Patients with multiple-site infection were counted in each applicable infection category.

Abbreviations: BMI, body mass index (kg/m^2^); GI, gastrointestinal; ICU, intensive care unit; IQR, interquartile range; SBP, spontaneous bacterial peritonitis; SSTI, skin and soft tissue infection; STSS, streptococcal toxic shock syndrome; UTI, urinary tract infection.

^a^Significant differences are observed.

Streptococcal toxic shock syndrome was diagnosed in 28 cases (6.3%), and polymicrobial bacteremia was observed in 66 cases (14.9%). The most common co-pathogen was *Staphylococcus aureus* (n = 26; methicillin-susceptible, n = 17; methicillin-resistant, n = 3; methicillin susceptibility unspecified, n = 6), followed by *Escherichia coli* (n = 13) and other *Streptococcus* species (n = 12). Overall, 7.3% of patients underwent percutaneous drainage, 13.2% underwent surgical drainage, and 22.3% required ICU admission. Compared with survivors, 30-day non-survivors were less likely to undergo surgical drainage (2.4% vs 14.2%, *P* = .03) and more likely to require ICU admission (51.2% vs 19.3%, *P* < .001).

### 30-Day Mortality

The 30-day mortality of GBS bacteremia was 9.3% (41/442), whereas the overall in-hospital mortality rate was 11.3% (50/442). Factors associated with 30-day mortality are shown in Table 2. In univariable analysis, hospital-acquired infection, 4 or more comorbidities, immunosuppressive therapy, heart failure, cirrhosis, advanced renal dysfunction, polymicrobial bacteremia, bacteremia without focus, respiratory infection, STSS, and ICU admission were significantly associated with 30-day mortality, whereas SSTI alone was associated with significantly lower mortality.

**Table 2.** Factors Associated With 30-Day Mortality Among Patients With Group B Streptococcus Bacteremia

Variable	Death, n/N (%)	Univariable OR (95% CI)	*P* Value	Multivariable OR (95% CI)	*P* Value
Age (per year increase)		1.02 (0.99–1.04)	.13	1.02 (0.99–1.05)	.12
Male sex		0.97 (0.51–1.85)	.93		
BMI		0.96 (0.89–1.03)	.23		
Hospital-acquired infection	12/59 (20.3%)	3.12 (1.49–6.52)	.003^a^		
Number of comorbidities ≥4	9/25 (36.0%)	6.77 (2.77–16.53)	<.001^a^		
Diabetes mellitus	19/169 (11.2%)	1.45 (0.76–2.76)	.26		
Solid tumor	14/126 (11.1%)	1.34 (0.68–2.64)	.40		
Hematological malignancy	3/32 (9.4%)	1.01 (0.29–3.48)	.98		
Chemotherapy	7/43 (16.3%)	2.09 (0.86–5.05)	.10		
Immunosuppressive therapy	8/32 (25.0%)	3.81 (1.59–9.14)	.003^a^	4.62 (1.66–12.86)	.003^a^
Dementia	5/51 (9.8%)	1.07 (0.40–2.87)	.890		
Heart failure	12/78 (15.4%)	2.10 (1.02–4.33)	.04^a^		
Cirrhosis	9/43 (20.9%)	3.04 (1.34–6.88)	.008^a^	3.40 (1.23–9.40)	.02^a^
Advanced renal dysfunction	11/43 (25.6%)	4.23 (1.94–9.22)	<.001^a^	2.66 (1.09–6.52)	.03^a^
Bacteremia without focus	17/120 (14.2%)	2.05 (1.06–3.97)	.03^a^		
SSTI	1/116 (0.9%)	0.06 (0.01–0.46)	.006^a^	0.07 (0.01–0.53)	.01^a^
UTI	11/96 (11.5%)	1.36 (0.66–2.83)	.41		
Musculoskeletal infection	6/81 (7.4%)	0.75 (0.30–1.84)	.52		
Cardiovascular infection	4/26 (15.4%)	1.86 (0.61–5.69)	.28		
Respiratory infection	6/23 (26.1%)	3.87 (1.43–10.45)	.008^a^	3.30 (1.08–10.06)	.04^a^
Abdominal infection	2/12 (16.7%)	2.01 (0.42–9.48)	.38		
STSS	11/28 (39.3%)	8.28 (3.56–19.27)	<.001^a^	9.16 (3.28–25.58)	<.001^a^
Polymicrobial bacteremia	15/66 (22.7%)	3.96 (1.97–7.97)	<.001^a^	2.40 (1.03–5.57)	.04^a^
Surgical drainage	1/58 (1.7%)	0.15 (0.02–1.12)	.06		
ICU admission	21/98 (21.4%)	4.38 (2.26–8.48)	<.001^a^		

Odds ratios (ORs) and 95% confidence intervals (CIs) were estimated using logistic regression analysis. Continuous variables (age and BMI) were analyzed per unit increase. Variables with *P* < .10 in univariable analysis and clinically relevant variables were included in the multivariable model.

Abbreviations: BMI, body mass index (kg/m^2^); CI, confidence interval; ICU, intensive care unit; SSTI, skin and soft tissue infection; STSS, streptococcal toxic shock syndrome; UTI, urinary tract infection.

^a^Significant differences are observed.

In multivariable analysis, immunosuppressive therapy (adjusted OR [aOR], 4.62; 95% confidence interval [CI], 1.66–12.86, *P* = .003), cirrhosis (aOR, 3.40; 95% CI, 1.23–9.40, *P* = .02), advanced renal dysfunction (aOR, 2.66; 95% CI, 1.09–6.52, *P* = .03), polymicrobial bacteremia (aOR, 2.40; 95% CI, 1.03–5.57, *P* = .04), respiratory infection (aOR, 3.30; 95% CI, 1.08–10.06, *P* = .04), and STSS (aOR, 9.16; 95% CI, 3.28–25.58, *P* < .001) remained independently associated with 30-day mortality. SSTI remained independently associated with lower mortality (aOR, 0.07; 95% CI, 0.01–0.53, *P* = .01) (Figure 2). In sensitivity analyses excluding polymicrobial bacteremia, these associations were materially unchanged (Supplementary Table 3). As shown in Figure 3, 30-day mortality tended to increase with age in the overall cohort, although this trend was not statistically significant (*P* = .11). In contrast, mortality remained high across all age groups among patients with STSS (28.6%–50.0%). Kaplan–Meier analysis demonstrated significant differences in 30-day survival across selected infection categories, including SSTI (1/116, 0.9%), bacteremia without focus (17/120, 14.2%), respiratory infection (6/23, 26.1%), and STSS (11/28, 39.3%) (log-rank *P* < .001) (Figure 4). Survival was highest among patients with SSTI and lowest among those with STSS. In a supplementary analysis, patients with pneumonia were more likely to be male, reside in a long-term care facility, have dementia, and have a lower BMI than those without pneumonia (Supplementary Table 4).

**Figure 2.**
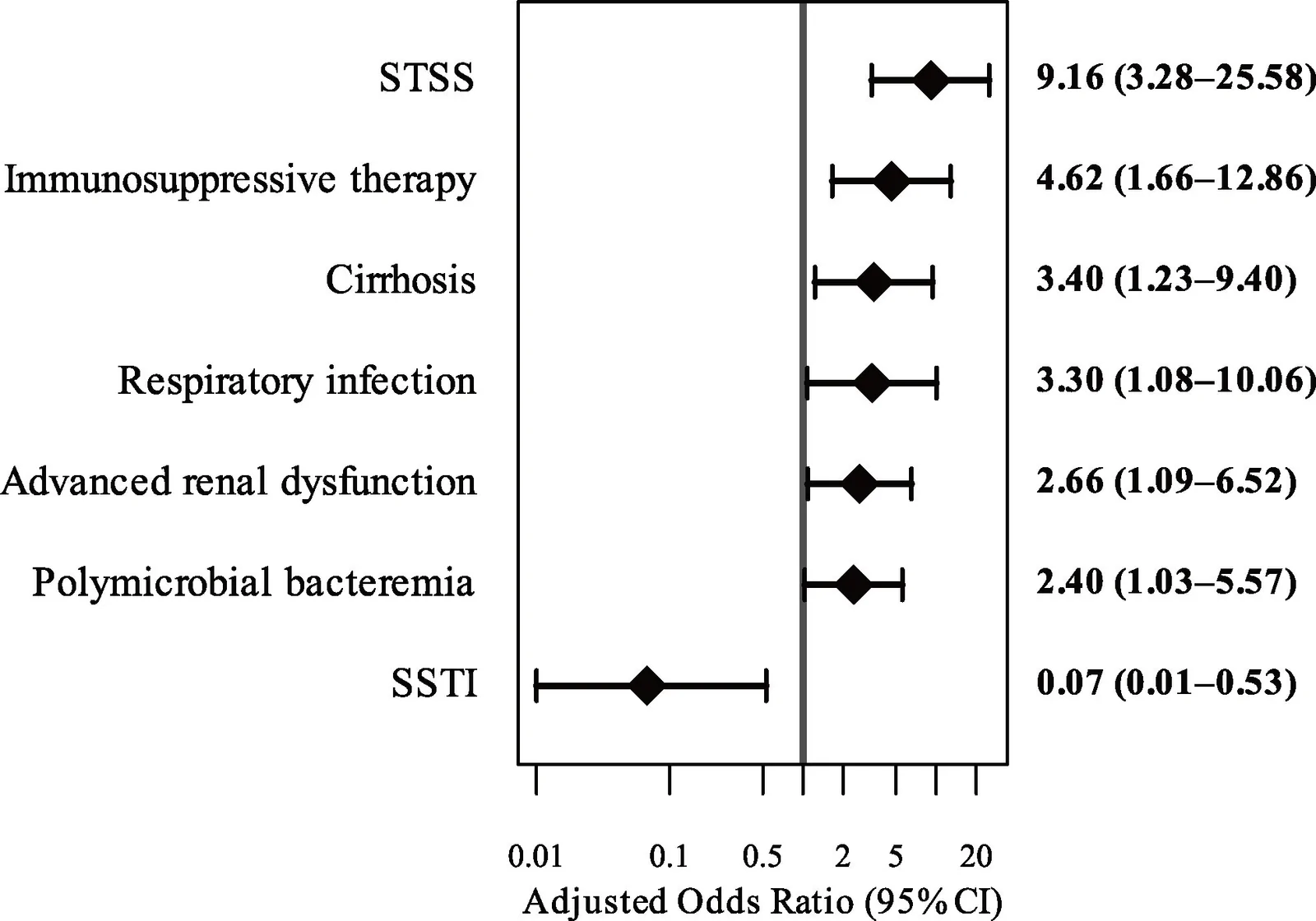
Forest plot of factors independently associated with 30-day mortality in patients with group B Streptococcus bacteremia. Abbreviations: SSTI, skin and soft tissue infection; STSS, streptococcal toxic shock syndrome.

**Figure 3.**
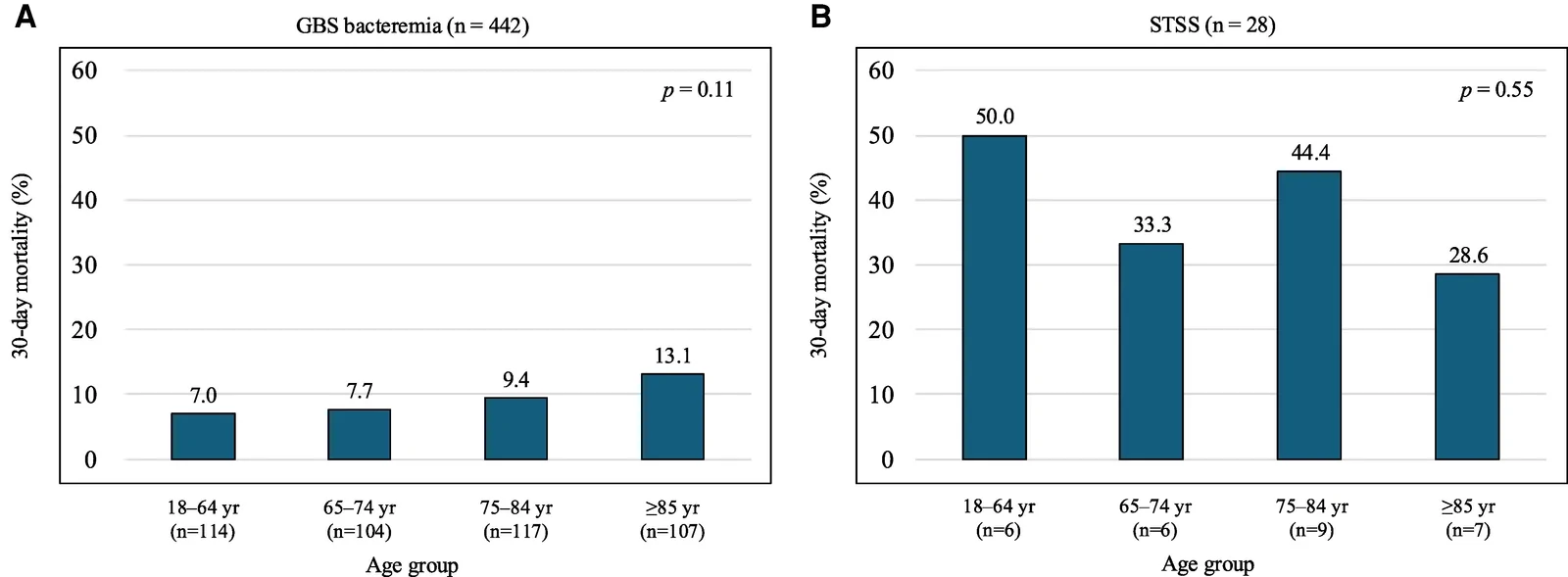
Age-stratified 30-day mortality among patients with group B Streptococcus bacteremia. Thirty-day mortality across age groups is shown for (A) the overall cohort of patients with group B Streptococcus (GBS) bacteremia and (B) the subgroup with streptococcal toxic shock syndrome (STSS). *P* values were calculated using the Cochran–Armitage trend test.

**Figure 4.**
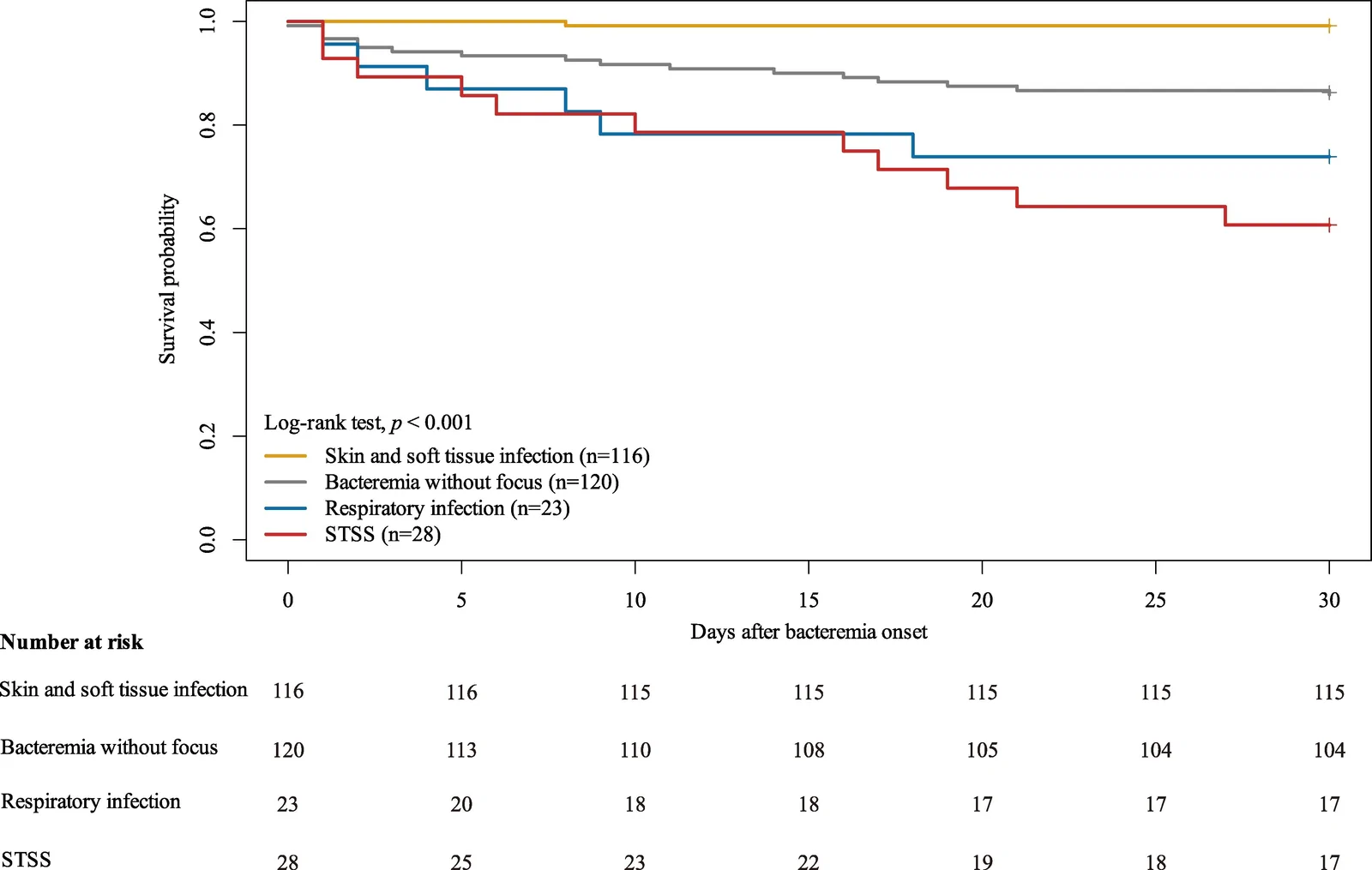
Kaplan–Meier curves for 30-day survival according to infectious pattern in patients with group B Streptococcus bacteremia. Thirty-day mortality was 0.9% (1/116) for skin and soft tissue infection, 14.2% (17/120) for bacteremia without focus, 26.1% (6/23) for respiratory infection, and 39.3% (11/28) for STSS. Abbreviation: STSS, streptococcal toxic shock syndrome.

### Streptococcal Toxic Shock Syndrome

The clinical characteristics of patients with STSS are presented in Supplementary Table 5. Baseline comorbidities were generally similar between patients with and without STSS. In contrast, patients with STSS had markedly higher rates of necrotizing fasciitis (14.3% vs 0.5%, *P* < .001), polymicrobial bacteremia (46.4% vs 12.8%, *P* < .001), ICU admission (85.7% vs 18.0%, *P* < .001), and 30-day mortality (39.3% vs 7.2%, *P* < .001).

### Antimicrobial Susceptibility and Definitive Therapy

The antimicrobial susceptibility profiles of the GBS isolates are presented in Table 3. They were highly susceptible to β-lactam antibiotics: penicillin G (434/438, 99.1%), ampicillin (436/438, 99.5%), ceftriaxone (297/298, 99.7%), cefepime (316/321, 98.4%), and meropenem (322/322, 100%). All tested isolates were susceptible to vancomycin (264/264, 100%) and linezolid (127/127, 100%). In contrast, reduced susceptibility was frequently observed for macrolides, clindamycin, and levofloxacin. The susceptibility rates were 66.8% (187/280) for erythromycin, 85.6% (369/431) for clindamycin, and 66.1% (292/442) for levofloxacin. Susceptibility rates did not differ significantly between survivors and non-survivors for any antimicrobial agent tested.

**Table 3.** Antimicrobial Susceptibility Profiles of Group B Streptococcus Isolates According to 30-Day Survival Status

	All Patients n/N (%)	Survivors n/N (%)	Non-survivors n/N (%)	*P* Value
Penicillin G	434/438 (99.1%)	393/397 (99.0%)	41/41 (100.0%)	1.00
Ampicillin	436/438 (99.5%)	395/397 (99.5%)	41/41 (100.0%)	1.00
Ceftriaxone	297/298 (99.7%)	267/268 (99.6%)	30/30 (100.0%)	1.00
Cefepime	316/321 (98.4%)	285/290 (98.3%)	31/31 (100.0%)	1.00
Meropenem	322/322 (100.0%)	291/291 (100.0%)	31/31 (100.0%)	
Vancomycin	264/264 (100.0%)	237/237 (100.0%)	27/27 (100.0%)	
Linezolid	127/127 (100.0%)	117/117 (100.0%)	10/10 (100.0%)	
Erythromycin	187/280 (66.8%)	170/252 (67.5%)	17/28 (60.7%)	.53
Clindamycin	369/431 (85.6%)	337/392 (86.0%)	32/39 (82.1%)	.48
Levofloxacin	292/442 (66.1%)	262/401 (65.3%)	30/41 (73.2%)	.39

Percentages were calculated using the number of isolates tested for each antimicrobial agent as the denominator. Susceptibility was interpreted according to CLSI M100, 35th edition (2025). *P* values were calculated by comparing susceptibility rates between survivors and non-survivors using Fisher's exact test.

Definitive antimicrobial regimens are summarized in Supplementary Table 6. The most common definitive therapies were ampicillin (140 [32.3%]), ampicillin/sulbactam (83 [19.2%]), ceftriaxone (48 [11.1%]), and penicillin G (30 [6.9%]). Other regimens were used less frequently. In crude comparisons, 30-day mortality was lower among patients who received ampicillin monotherapy (3.6%, *P* = .01). No significant differences in mortality were observed among patients treated with ampicillin/sulbactam, ceftriaxone, or penicillin G. Among patients with STSS, definitive antimicrobial regimens were heterogeneous, most commonly ampicillin (n = 5) and ampicillin/sulbactam (n = 4); clindamycin was used in 2 cases.

## DISCUSSION

In this multicenter cohort of 442 adults with GBS bacteremia, the overall 30-day mortality was 9.3%. Development of STSS was the strongest independent predictor of death (aOR 9.16). Immunosuppressive therapy, followed by cirrhosis, advanced renal dysfunction, respiratory infection, and polymicrobial bacteremia, were also independently associated with a higher risk of 30-day mortality. Hospital-acquired infection accounted for 13.3% of the cases and was associated with higher 30-day mortality than community-acquired infection. In addition, mortality was significantly higher among patients with multimorbidity.

Invasive GBS disease predominantly affects older adults with underlying comorbidities [7, 19, 20]. Our cohort was similarly characterized by advanced age (including residents in long-term care facility: 10.4%) and a high burden of underlying multiple comorbidities, most commonly diabetes mellitus, malignancy, and heart failure. Bacteremia without focus (27.1%) was the most common clinical presentation, followed by SSTI (26.2%), UTI (21.7%), and musculoskeletal infection (18.3%); together, these accounted for approximately 90% of all cases. The relatively high frequency of UTI is also consistent with findings from a recent Japanese cohort of older adults [7].

The mortality rate of GBS bacteremia observed in this study aligns with previously reported figures, ranging from 5% to 13% [19–22]. Notably, patient prognosis varied markedly according to the site of infection. Mortality was as low as 0.9% (1/116) in patients with SSTIs, whereas it reached 26.1% (6/23) in those with respiratory infections. Our findings are broadly consistent with those of a large US Veterans Health Administration cohort, which showed that mortality varied substantially across invasive GBS diseases, with higher mortality in pneumonia or empyema (17.5%) and lower mortality in SSTI (3.5%) and musculoskeletal infections (2.0%) [19]. Despite differences in study populations—specifically their inclusion of all invasive GBS cases and a predominantly male veteran cohort—both studies consistently demonstrate that prognosis diverges substantially according to the infection site. The higher mortality associated with respiratory infections may be partly explained by their tendency to occur in older, debilitated adults, particularly those with dementia or neurologic impairment, in whom aspiration may contribute to disease development [19, 23, 24]. In our cohort, patients with pneumonia had significantly lower BMI and were more likely to reside in long-term care facilities and have dementia, suggesting a potentially frailer population with features associated with aspiration risk. However, because aspiration and frailty were not directly assessed, this interpretation remains hypothetical. Conversely, the favorable prognosis observed in patients with SSTIs may stem from earlier clinical recognition, prompt initiation of appropriate antimicrobial therapy, and the feasibility of timely source control.

Overall comorbidity burden would also be an important determinant of prognosis. A multicenter study from Brussels identified Charlson Comorbidity Index (CCI) ≥4 as an independent risk factor for mortality among nonpregnant adults with invasive GBS diseases [25]. In our cohort, although CCI was not evaluated, patients with 4 or more comorbidities had significantly higher mortality (36.0%). Among individual comorbid conditions, cirrhosis, advanced renal dysfunction, and immunosuppressive therapy were independently associated with 30-day mortality. The association with advanced renal dysfunction is consistent with previous reports identifying chronic kidney disease as a poor prognostic factor in adults with invasive GBS disease [26], whereas data specifically linking cirrhosis and immunosuppressive therapy to mortality in GBS bacteremia remain limited. These findings suggest that both overall comorbidity burden and specific underlying diseases potentially contribute to poor outcomes in GBS bacteremia.

STSS is a fulminant septic shock syndrome characterized by abrupt onset and rapid progression and is a nationally notifiable disease in Japan. Previous nationwide surveillance studies have reported mortality rates of approximately 25%–56% among nonpregnant adults with GBS-associated STSS [10, 16]. The 30-day mortality among patients with GBS-associated STSS in our cohort was 39.3% (11/28), which was within the range reported in previous studies, and STSS was the strongest independent predictor of death in our multivariable analysis. Previous studies have reported inconsistent findings regarding the association between age and mortality in GBS-associated STSS [10, 16]. In our cohort, mortality remained high across all age groups, with no clear age-related gradient.

Risk factors predisposing patients to GBS-associated STSS remain incompletely understood [11]. In our cohort, baseline characteristics did not differ significantly between patients with and without STSS. The lack of clear associations between STSS and specific baseline comorbidities suggests that host vulnerability alone does not fully explain its poor prognosis. Thus, pathogen-related factors would primarily contribute to the development and severity of STSS. Previous studies have reported that serotype Ib is the most frequent among GBS isolates associated with STSS, followed by serotype V [10]. Nevertheless, given that these serotypes are commonly isolated across the spectrum of invasive GBS diseases [14], a specific genetic determinant remains to be elucidated as a reliable marker for the development of STSS [27]. Together, these facts suggest that the pathogenesis of STSS is likely multifactorial, involving both host and pathogen determinants, and further studies integrating clinical and molecular epidemiologic data are warranted.

Regarding antimicrobial susceptibility, GBS isolates remained highly susceptible to β-lactam agents, supporting penicillin or ampicillin as first-line therapy. In contrast, resistance to macrolides, clindamycin, and levofloxacin has been increasingly common, underscoring the importance of susceptibility testing when alternative agents are considered. Fluoroquinolone resistance in GBS has been associated with serotype Ib strains belonging to clonal complex 10 [14]. A previous surveillance study from Japan reported a fluoroquinolone resistance rate of 33.9%, similar to that observed in our cohort [14]. Data from the Japan Nosocomial Infections Surveillance (JANIS) showed that the frequency of penicillin-resistant GBS isolates from various clinical sources ranged from 5.3% to 6.8% during 2015–2024 [28]. In contrast, the prevalence of penicillin-resistant GBS isolates in our cohort was notably low at 1.0%. This divergence from previous reports may be attributed to differences in specimen sources—given that our study focused exclusively on blood culture isolates—and variations in susceptibility testing methodologies. Continued surveillance of penicillin resistance among invasive GBS isolates remains warranted. In our crude analyses, ampicillin monotherapy was associated with lower mortality. However, this finding likely reflects a selection bias toward less severely ill patients rather than a true therapeutic advantage. Given the observational nature of this study, the comparative effectiveness of different antibiotic regimens remains to be determined.

This study has several limitations to be addressed. First, the retrospective design precludes causal inference, and residual confounding factors may remain despite multivariable adjustment. In addition, illness severity and comorbidity burden at the onset of GBS bacteremia could not be fully assessed because data required to calculate measures such as the SOFA score, APACHE score, and CCI were not systematically available. Second, our data may not fully represent all invasive GBS diseases, as cases with GBS isolation from sterile sites other than blood were not captured. Third, infection sites were determined retrospectively at each participating institution and were not centrally adjudicated, which may have introduced misclassification. Fourth, the participating hospitals were exclusively located in western Japan, which may limit the generalizability of our findings to other regions. Fifth, the absence of serotype and molecular data precluded a detailed assessment of strain-specific virulence factors. Antimicrobial susceptibility data were incomplete because testing panels varied across institutions, and inducible clindamycin resistance was not systematically assessed. In addition, differences in susceptibility testing platforms across institutions and the lack of centralized confirmatory testing may have introduced interlaboratory variability. Finally, the relatively small number of deaths may have constrained model stability, and data on the timeliness of appropriate therapy and adequacy of source control were not systematically available. Nonetheless, this study represents one of the largest multicenter analyses of adult GBS bacteremia in Japan so far, providing a comprehensive clinical characterization across a diverse range of healthcare institutions.

## CONCLUSION

In conclusion, the adult GBS bacteremia predominantly affected older patients with multiple comorbidities and most commonly presented as bacteremia without focus, SSTI, UTI, or musculoskeletal infection. Thirty-day mortality was independently associated with immunosuppression, cirrhosis, advanced renal dysfunction, respiratory tract infection, polymicrobial bacteremia, and particularly STSS. STSS was associated with high mortality across all adult age groups.

## Supplementary Material

ofag665_Supplementary_Data

## Supplementary Data

Supplementary materials are available at *Open Forum Infectious Diseases* online. Consisting of data provided by the authors to benefit the reader, the posted materials are not copyedited and are the sole responsibility of the authors, so questions or comments should be addressed to the corresponding author.
