# Supplementary material for: Clinical Characteristics and Mortality Predictors of Group B Streptococcus Bacteremia in Adults: A Multicenter Retrospective Cohort Study in Japan, 2015–2024
Source: Open Forum Infect Dis. 2026 Oct 9;13(10):ofag665. doi: 10.1093/ofid/ofag665 (PMC13684403; doi:10.1093/ofid/ofag665)
Supplement: Supplementary file 1 [file ofag665_supplementary_data.docx]

**Supplementary Table 1. Characteristics of the participating hospitals and numbers of group B Streptococcus (GBS) bacteremia cases.**

| **Participating hospitals** | **Number of beds** | **Full-time infectious disease specialist available for consultation** | **Antimicrobial susceptibility testing platform** | **Microbiological identification method** | **All GBS bacteremia cases during the study period** | **Enrolled cases** | **30-day mortality (%)** |
| --- | --- | --- | --- | --- | --- | --- | --- |
| Kurashiki Central Hospital  (Okayama Prefecture) | 1,172 | No | MicroScan WalkAway system | MALDI Biotyper | 162 | 135 | 8.9% |
| Okayama University Hospital  (Okayama Prefecture) | 819 | Yes | Dry Plate ‘Eiken’ | MALDI Biotyper | 48 | 43 | 4.7% |
| Japanese Red Cross Society Himeji Hospital  (Hyogo Prefecture) | 560 | No | MicroScan WalkAway system | MicroScan WalkAway system | 88 | 63 | 4.8% |
| Shimane Prefectural Central Hospital  (Shimane Prefecture) | 544 | Yes | VITEK 2 system | VITEK 2 system until June 2019; MALDI Biotyper thereafter | 59 | 51 | 11.8% |
| Tottori Prefectural Central Hospital  (Tottori Prefecture) | 511 | Yes | MicroScan WalkAway system until November 2018; BD Phoenix M50 system thereafter | MicroScan WalkAway system until November 2018; BD Phoenix M50 system thereafter | 65 | 49 | 10.2% |
| Tsuyama Chuo Hospital  (Okayama Prefecture) | 456 | Yes | MicroScan WalkAway system | MicroScan WalkAway system until October 2017; MALDI Biotyper thereafter | 94 | 73 | 15.1% |
| Masuda Red Cross Hospital  (Shimane Prefecture) | 284 | No | Disk diffusion method until March 2020; MicroScan WalkAway system thereafter | MicroScan WalkAway system | 31 | 28 | 7.1% |
| Total |  |  |  |  | 547 | 442 | 9.3% |

Manufacturers were as follows: MicroScan WalkAway system, Beckman Coulter; Dry Plate ‘Eiken’, Eiken Chemical; VITEK 2 system, bioMérieux; BD Phoenix M50 system, Becton Dickinson; and MALDI Biotyper, Bruker Daltonics.

**Supplementary Table 2. Detailed distribution of infection sites among 442 adults with group B Streptococcus bacteremia.**

| **Bacteremia without focus** | 120 (27.1%) | Septic arthritis | 32 (7.2%) | **Respiratory infection** | 23 (5.2%) |
| --- | --- | --- | --- | --- | --- |
| **Skin and soft tissue infection** | 116 (26.2%) | knee | 16 (3.6%) | Pneumonia | 22 (5.0%) |
| Cellulitis | 95 (21.5%) | shoulder | 5 (1.1%) | Pleuritis | 1 (0.2%) |
| Diabetic foot infection | 8 (1.8%) | hip | 4 (0.9%) | **Abdominal infection** | 12 (2.7%) |
| Necrotizing fasciitis | 6 (1.4%) | sacroiliac | 3 (0.7%) | Spontaneous bacterial peritonitis | 3 (0.7%) |
| Pressure ulcer infection | 4 (0.9%) | metacarpophalangeal | 1 (0.2%) | Enteritis | 3 (0.7%) |
| Burn wound infection | 1 (0.2%) | acromioclavicular | 1 (0.2%) | Pelvic inflammatory disease | 2 (0.5%) |
| Surgical site infection | 1 (0.2%) | costovertebral | 1 (0.2%) | Gastrointestinal perforation | 1 (0.2%) |
| Inguinal lymphadenitis | 1 (0.2%) | unspecified | 2 (0.5%) | Cholecystitis | 1 (0.2%) |
| **Urinary tract infection** | 96 (21.7%) | Iliopsoas abscess | 8 (1.8%) | Cholangitis | 1 (0.2%) |
| Pyelonephritis | 93 (21.0%) | Epidural abscess | 8 (1.8%) | Liver abscess | 1 (0.2%) |
| Renal abscess | 1 (0.2%) | Osteomyelitis | 4 (0.9%) | Retroperitoneal abscess | 1 (0.2%) |
| Prostatitis | 1 (0.2%) | Sacroiliac joint abscess | 1 (0.2%) | **Others** | 12 (2.7%) |
| Prostatic abscess | 1 (0.2%) | Piriformis abscess | 1 (0.2%) | Meningitis | 5 (1.1%) |
| **Musculoskeletal infection** | 81 (18.3%) | **Cardiovascular infection** | 26 (5.9%) | Endophthalmitis | 2 (0.5%) |
| Spondylitis | 39 (8.8%) | Infective endocarditis | 20 (4.5%) | Chorioamnionitis | 1 (0.2%) |
| cervical spine | 7 (1.6%) | Infected aneurysm | 2 (0.5%) | Endometritis | 1 (0.2%) |
| thoracic spine | 6 (1.4%) | Pericarditis | 2 (0.5%) | Tonsillitis | 1 (0.2%) |
| lumber spine | 30 (6.8%) | Vascular graft infection | 2 (0.5%) | Mediastinal abscess | 1 (0.2%) |
|  |  | Aortic annular abscess | 1 (0.2%) | Device-related infection | 1 (0.2%) |

Percentages were calculated using the total cohort of 442 patients. Some patients had more than one infectious focus within a major category; therefore, the sum of subcategories may exceed the number of patients in that category.

**Supplementary Table 3. Sensitivity analysis excluding polymicrobial bacteremia from the multivariable model.**

| **Variable** | **Adjusted OR (95% CI)** | ***p* value** |
| --- | --- | --- |
| Age (per year increase) | 1.02 (0.99–1.05) | 0.14 |
| Immunosuppressive therapy | 4.88 (1.78–13.38) | 0.002 |
| Cirrhosis | 2.97 (1.09–8.09) | 0.03 |
| Advanced renal dysfunction | 2.78 (1.13–6.81) | 0.03 |
| SSTI | 0.06 (0.01–0.50) | 0.009 |
| Respiratory infection | 4.17 (1.43–12.22) | 0.009 |
| STSS | 12.00 (4.45–32.40) | <0.001 |

Adjusted odds ratios were estimated using multivariable logistic regression after excluding polymicrobial bacteremia from the model. OR, odds ratio; CI, confidence interval; SSTI, skin and soft tissue infection; STSS, streptococcal toxic shock syndrome.

**Supplementary Table 4. Clinical characteristics of patients with and without pneumonia.**

|  | Non-pneumonia  (n = 420) | Pneumonia  (n = 22) | *p* value |
| --- | --- | --- | --- |
| Age (years), median [IQR] | 75.0 [64–84] | 80.5 [67–85] | 0.31 |
| Male sex | 240 (54.3%) | 18 (81.8%) | 0.008 |
| BMI, median [IQR] | 22.5 [19.9–25.9] | 19.2 [16.6–23.0] | 0.008 |
| Residence in long-term care facility | 39 (9.3%) | 7 (31.8%) | 0.004 |
| Hospital-acquired infection | 57 (13.6%) | 2 (9.1%) | 0.75 |
| Number of comorbidities |  |  |  |
| 0 | 71 (16.9%) | 4 (18.2%) | 0.78 |
| 1 | 153 (36.4%) | 5 (22.7%) | 0.26 |
| 2–3 | 174 (41.4%) | 10 (45.5%) | 0.83 |
| ≥4 | 22 (5.2%) | 3 (13.6%) | 0.12 |
| Comorbidities |  |  |  |
| Obesity (BMI ≥30) | 29 (7.2%) | 1 (5.6%) | 1.00 |
| Diabetes mellitus | 161 (38.3%) | 8 (36.4%) | 1.00 |
| History of malignancy |  |  |  |
| Solid tumor | 120 (28.6%) | 6 (27.3%) | 1.00 |
| Hematological malignancy | 31 (7.4%) | 1 (4.5%) | 1.00 |
| Chemotherapy | 40 (9.5%) | 3 (13.6%) | 0.46 |
| Immunosuppressive therapy | 31 (7.4%) | 1 (4.5%) | 1.00 |
| Stroke | 51 (12.1%) | 5 (22.7%) | 0.18 |
| Dementia | 44 (10.5%) | 7 (31.8%) | 0.008 |
| Heart failure | 74 (17.6%) | 4 (18.2%) | 1.00 |
| Cirrhosis | 41 (9.8%) | 2 (9.1%) | 1.00 |
| Advanced renal dysfunction | 39 (9.3%) | 4 (18.2%) | 0.25 |
| Pregnancy | 1 (0.2%) | 0 (0%) | 1.00 |
| Multiple-site infection | 40 (9.5%) | 2 (9.1%) | 1.00 |
| STSS | 27 (6.4%) | 1 (4.5%) | 1.00 |
| Polymicrobial bacteremia | 57 (13.6%) | 4 (40.9%) | 0.002 |
| ICU admission | 92 (22.1%) | 6 (27.3%) | 0.60 |
| 30-day mortality | 36 (8.6%) | 5 (22.7%) | 0.04 |

Data are presented as number (%) for categorical variables and median (interquartile range) for continuous variables. P values were calculated using the Fisher exact test for categorical variables and the Mann–Whitney U test for continuous variables. Abbreviations: BMI, body mass index (kg/m²); IQR, interquartile range; STSS, streptococcal toxic shock syndrome; ICU, intensive care unit.

**Supplementary Table 5. Clinical characteristics of patients with and without streptococcal toxic shock syndrome (STSS).**

|  | Non-STSS  (n = 414) | STSS  (n = 28) | *p* value |
| --- | --- | --- | --- |
| Age (years), median [IQR] | 75.0 [64–84] | 75.0 [69–84] | 0.65 |
| Male sex | 227 (54.8%) | 13 (46.4%) | 0.44 |
| BMI, median [IQR] | 22.3 [19.8–25.7] | 23.1 [19.8–26.9] | 0.80 |
| Residence in long-term care facility | 42 (10.1%) | 4 (14.3%) | 0.52 |
| Hospital-acquired infection | 56 (13.5%) | 3 (10.7%) | 1.00 |
| Number of comorbidities |  |  |  |
| 0 | 72 (17.4%) | 3 (10.7%) | 0.45 |
| 1 | 150 (36.2%) | 8 (28.6%) | 0.54 |
| 2–3 | 170 (41.1%) | 14 (50.0%) | 0.43 |
| ≥4 | 22 (5.3%) | 3 (10.7%) | 0.20 |
| Comorbidities |  |  |  |
| Obesity (BMI ≥30) | 28 (7.1%) | 2 (7.7%) | 0.71 |
| Diabetes mellitus | 156 (37.7%) | 13 (46.4%) | 0.42 |
| History of malignancy |  |  |  |
| Solid tumor | 119 (28.7%) | 7 (25.0%) | 0.83 |
| Hematological malignancy | 32 (7.7%) | 0 (0%) | 0.25 |
| Chemotherapy | 41 (9.9%) | 2 (7.1%) | 1.00 |
| Immunosuppressive therapy | 30 (7.2%) | 2 (7.1%) | 1.00 |
| Stroke | 51 (12.3%) | 5 (17.9%) | 0.38 |
| Dementia | 47 (11.4%) | 4 (14.3%) | 0.55 |
| Heart failure | 71 (17.1%) | 7 (25.0%) | 0.31 |
| Cirrhosis | 38 (9.2%) | 5 (17.9%) | 0.18 |
| Advanced renal dysfunction | 39 (9.4%) | 4 (14.3%) | 0.34 |
| Pregnancy | 1 (0.2%) | 0 (0%) | 1.00 |
| Site of infection |  |  |  |
| Bacteremia without focus | 113 (27.3%) | 7 (25.0%) | 1.00 |
| SSTI | 108 (26.1%) | 8 (28.6%) | 0.82 |
| Cellulitis | 91 (22.0%) | 4 (14.3%) | 0.48 |
| Diabetic foot | 8 (1.9%) | 0 (0%) | 1.00 |
| Necrotizing fasciitis | 2 (0.5%) | 4 (14.3%) | <0.001 |
| UTI | 89 (21.5%) | 7 (25.0%) | 0.64 |
| Pyelonephritis | 86 (20.8%) | 7 (25.0%) | 0.63 |
| Musculoskeletal infection | 78 (18.8%) | 3 (10.7%) | 0.45 |
| Spondylitis | 39 (9.4%) | 0 (0%) | 0.16 |
| Septic arthritis | 30 (7.2%) | 2 (7.1%) | 1.00 |
| Cardiovascular infection | 23 (5.6%) | 3 (10.7%) | 0.22 |
| Infective endocarditis | 17 (4.1%) | 3 (10.7%) | 0.13 |
| Respiratory infection | 22 (5.3%) | 1 (3.6%) | 1.00 |
| Pneumonia | 21 (5.1%) | 1 (3.6%) | 1.00 |
| Pleuritis | 1 (0.2%) | 0 (0%) | 1.00 |
| Abdominal infection | 10 (2.4%) | 2 (7.1%) | 0.17 |
| SBP | 2 (0.5%) | 1 (3.6%) | 0.18 |
| GI perforation | 1 (0.2%) | 0 (0%) | 1.00 |
| Others | 12 (2.9%) | 0 (0%) | 1.00 |
| Multiple-site infection | 39 (9.4%) | 3 (10.7%) | 0.74 |
| Polymicrobial bacteremia | 53 (12.8%) | 13 (46.4%) | <0.001 |
| Percutaneous drainage | 28 (6.8%) | 4 (14.3%) | 0.14 |
| Surgical drainage | 51 (12.3%) | 7 (25.0%) | 0.08 |
| ICU admission | 74 (18.0%) | 24 (85.7%) | <0.001 |
| 30-day mortality | 30 (7.2%) | 11 (39.3%) | <0.001 |

Data are presented as number (%) for categorical variables and median (interquartile range) for continuous variables. P values were calculated using the Fisher exact test for categorical variables and the Mann–Whitney U test for continuous variables. Abbreviations: BMI, body mass index (kg/m²); IQR, interquartile range; SSTI, skin and soft tissue infection; UTI, urinary tract infection; SBP, spontaneous bacterial peritonitis; GI, gastrointestinal; STSS, streptococcal toxic shock syndrome; ICU, intensive care unit.

**Supplementary Table 6. Definitive antimicrobial therapy and 30-day mortality.**

|  | Overall, n (%) | 30-day mortality, n/N (%) | *p* value |
| --- | --- | --- | --- |
| Ampicillin | 140 (32.3%) | 5/140 (3.6%) | 0.01 |
| Ampicillin/sulbactam | 83 (19.2%) | 10/83 (12.0%) | 0.18 |
| Ceftriaxone | 48 (11.1%) | 5/48 (10.4%) | 0.58 |
| Penicillin G | 30 (6.9%) | 2/30 (6.7%) | 1.00 |
| Others | 132 (30.5%) | 14/132 (10.6%) | 0.26 |

Nine patients with no definitive therapy or missing definitive treatment data were excluded from this analysis. Percentages in the Overall column were calculated using 433 patients with evaluable definitive therapy data as the denominator. Others included less frequently used single-agent regimens and combination regimens. P values were calculated using Fisher’s exact test.
